# NRP1 and MMP9 are dual targets of RNA‐binding protein QKI5 to alter VEGF‐R/ NRP1 signalling in trophoblasts in preeclampsia

**DOI:** 10.1111/jcmm.16580

**Published:** 2021-05-04

**Authors:** Xingyu Yang, Dan Chen, Biwei He, Weiwei Cheng

**Affiliations:** ^1^ The International Peace Maternity and Child Health Hospital School of Medicine Shanghai Jiao Tong University Shanghai China; ^2^ Shanghai Key Laboratory of Embryo Original Diseases Shanghai China; ^3^ Shanghai Municipal Key Clinical Specialty Shanghai China

**Keywords:** MMP9, NRP1, preeclampsia, QKI5, trophoblast

## Abstract

Preeclampsia (PE) is characterized by placental ischemia and hypoxia, resulting in abnormal casting of the uterine spiral artery, which is mainly caused by insufficient trophoblastic cell infiltration. A reduction in levels of growth factor‐based signalling via Neuropilin‐1 (NRP1) has been shown to contribute to dysfunctional trophoblast development. In this study, we showed that the RNA‐binding protein, QKI5, regulated NRP1 expression and significantly improved trophoblast proliferation in vitro and in vivo. QKI5 and NRP1 expressions were significantly reduced in human PE placentas and in trophoblasts during hypoxia. Overexpression of these factors significantly improved cell proliferation and migration in vitro, in contrast to a decrease upon siRNA knockdown of QKI5 and NRP1 in HTR‐8/SVneo cells. Using RIP and RNA pull‐down assays, we further showed that QKI5 directly interacted with the 3'‐UTR region of NRP1, to mediate cell proliferation and migration via matrix metalloprotease‐9. Further, similar to NRP1, QKI5 also targets matrix metalloproteinase 9 (MMP9) involved in secretion of growth factors and its effects can be counteracted by NRP1 overexpression. In vivo studies using a PE mouse model revealed that QKI5 overexpression alleviated PE‐related symptoms such as elevated blood pressure and proteinuria. Taken together, we found that QKI5 was a novel regulator, of VEGF‐R/NRP1 signalling pathway functioning in trophoblast proliferation and migration, resulting in major contributors to the pathogenesis of PE. While careful evaluation of the broad implications of QKI5 expression is still necessary, this study identified QKI5 as a promising target for treatment strategies in acute PE patients.

## 1 INTRODUCTION

Preeclampsia (PE) is a severe obstetrical hypertension‐related complication characterized by high blood pressure and increased levels of protein in the urine (proteinuria).^1^ It is one of the major causes of mortality in pregnant women, responsible for approximately 18% of all pregnancy‐related deaths.^2^, ^3^ PE is a multifactorial disorder and broad‐spectrum antihypertensive drugs cannot be used for treatment due to severe neonatal side effects.^4^, ^5^ Progression of PE involves oxidative stress, induction of systemic inflammation and release of angiogenic factors into the circulatory system.^6^, ^7^, ^8^ This results in placental damage due to prolonged hypoxic conditions that trigger apoptosis‐induced cell death in trophoblasts.^9^, ^10^, ^11^ Prolonged hypoxia causes irreversible changes in cells including transcriptional reprogramming and regulation of gene expression that in turn affects intrinsic cell properties towards development and migration. Understanding these mechanisms in the context of PE remains key towards identifying alternative treatment strategies.

Several factors regulate gene expression including microRNAs, long non‐coding (lnc) RNAs and RNA‐binding proteins. Quaking homolog KH domain RNA‐binding (QKI) protein, belonging to the Signal Transduction and Activation of RNA (STAR) family of proteins, has been known to play a role in altering cellular differentiation processes in myeloid cells.^12^, ^13^ Under hypoxia, QKI directly regulated hypoxia‐induced factor‐1a (HIF‐1α) expression to support tumorigenesis.^14^ QKI isoforms have also been implicated in altering vascular endothelial growth factor receptor (VEGF‐R)/ Neuropilin‐1 (NRP1) co‐receptor signalling pathway in endothelial cell differentiation and functions.^15^, ^16^ They can compete with microRNAs to inhibit gene expression of NRP‐1/2 that are essential for early cell development.^13^ More specifically, QKI isoform 5 (QKI5) has been shown to play a role in altering stem cell differentiation and RNA‐splicing events in cancer.^17^, ^18^ It regulates key differentiation pathways during erythropoiesis, osteoclastogenesis and angiogenesis.^15^ However, QKI proteins and their possible regulatory roles in trophoblast differentiation nor dysregulation in the context of PE have not been elucidated so far.

Placental trophoblasts ensure differentiation via an interplay between different growth factor signalling pathways including VEGF‐R/NRP1 and are strictly controlled by regulators of gene expression.^19^, ^20^ Analysis of placental tissue samples from PE patients revealed that both NRP1 expression and VEGF‐R‐binding protein VEGF expression were significantly decreased;^21^ however, the mechanism behind its regulation remains elusive. VEGF signalling requires its secretion from extracellular matrices that are mediated by a specific class of proteinases called matrix metalloproteinases (MMPs). MMPs are capable of degrading the extracellular matrix proteins including collagen and fibronectin to facilitate release of growth factors required for cell growth and differentiation.^22^ MMPs have also been studied in the context of PE for their role in altering tissue architecture and promoting trophoblast invasion.^23^ MMP2 and MMP9 are implicated in the release of VEGF that is required for placental growth and uterine remodelling during the early stages of pregnancy.^24^ MMPs are up‐regulated during tumorigenesis to promote cell proliferation and invasion in a VEGF‐NRP1‐dependent manner.^25^, ^26^ In contrast, lower expression of MMPs is associated with various pregnancy‐related complications, including PE, by inducing apoptosis of trophoblasts and maternal intolerance.^27^, ^28^, ^29^ Although the role of MMPs has been broadly described in the context of trophoblast development and invasion, identifying their mechanism of regulation will lead to the development of efficient treatment alternatives.

In the present study, we showed that QKI5 was a novel regulator of NRP1 and MMP9 expression in trophoblasts during hypoxia. In addition to studying its effects in vitro in the HTR‐8/SVneo trophoblast cell line, we used an in vivo PE mouse model to evaluate the effects of QKI5 expression in regulating PE symptoms during pregnancy. Our findings identified QKI5‐mediated regulation of NRP1 expression as an important pathway in trophoblast development.

## 2 METHODS

### 2.1 Placental tissue collection

Human placental tissues were obtained from 20 patients during post‐normotensive pregnancy and from 20 patients who were prediagnosed with PE. Diagnosis for PE was in accordance with the criteria established by the National High Blood Pressure Education Working Group (Shanghai, China). Samples were obtained after written informed consent from the participants, and all procedures were approved by the Ethics Board of The International Peace Maternity and Child Health Hospital (Shanghai, China). The clinical informations from control and patients are recorded in Table 1.

**TABLE 1:** clinical informations of the study population

Characteristic	Normal (n = 20)	Preeclampsia (n = 20)	*P* value
Age of woman (years)	30.6 ± 0.8	31.1 ± 1	>.05
Body mass index (BMI; kg/m^2^)	27.4 ± 0.5	30.2 ± 0.9	<.01
Gestational age at delivery (weeks)	38.7 ± 0.2	35.5 ± 1	<.01
Systolic blood pressure (mmHg)	110.7 ± 23.15	169.5 ± 28.11	<.01
Diastolic blood pressure (mmHg)	75.6 ± 12.78	101.3 ± 14.6	<.01
Data are presented as mean ± SD			

### 2.2 Cell culture and transfection

In vitro experiments were conducted using the HTR‐8/SVneo human villous trophoblast cell line (Bio Life Technologies, Beijing, China), which were cultured in RPMI 1640 medium (Nacalai Tesque) supplemented with 5% foetal bovine serum (FBS) (Sigma‐Aldrich), 100 U/mL penicillin and 100 mg/mL streptomycin (Nacalai Tesque) at 37°C/5% CO_2_. Conditions for hypoxia treatment involved 1%–2% oxygen for 48 h as compared to 20% oxygen under normal conditions. Plasmids and siRNA transfections were performed with Lipofectamine 2000 (Life Technologies) according to the manufacturer's protocol. Briefly, QKI5 or NRP1 expression plasmids (pcDNA vector purchased from Addgene), or QKI5 siRNA or NRP1 (siRNA#1 107267, siRNA#2 107269 purchased from Thermo Fisher) were mixed with OptiMEM and added together with Lipofectamine in a 1:3 ratio. Two siRNAs (si‐QKI5#1 and si‐QKI5#2) specifically for QKI5 and the control siRNAs (si‐NC) were synthesized by Dharmacon as previously described.^30^ The DNA‐Lipofectamine complex was added to cells, and a fresh medium exchange was conducted after 6 h. The cells were then used 24 h post‐transfection.

### 2.3 Western blotting

Protein expression was analysed by harvesting cells in RIPA lysis buffer containing freshly added 1% phenylmethylsulfonyl fluoride for inhibiting proteases. The total protein content of cell lysates was estimated using the standard bicinchoninic acid (BCA) method (Abcam). Forty μg of protein per sample was denatured and loaded onto a 10% SDS‐PAGE gel and subjected to electrophoresis at 100 V for 1.5 h. The separated protein bands were transferred to a polyvinylidene difluoride membrane by semi‐dry blotting and probed with primary antibodies against QKI5 (anti‐human QKI5, 1:1,000, AB9904, Millipore), NRP1 (anti‐human NRP1, 1:1,000, ab81321, Abcam) or MMP9 (anti‐human MMP9, 1:2,000, ab38898, Abcam). β‐actin (anti‐human β‐actin, 1:1,000, ab8226, Abcam) or glyceraldehyde 3‐phosphate dehydrogenase (GAPDH) (anti‐human GAPDH, 1:3,000, ab181602, Abcam) were used as loading controls. Membranes were incubated with primary antibodies overnight at 4°C and further incubated with the corresponding horseradish peroxidase‐conjugated secondary antibodies, goat anti‐rabbit IgG‐HRP (1:10 000, ab6721) for 1 h at room temperature. Immunodetection of proteins using chemiluminescence was performed with Image‐Pro Plus 6.0 (Media Cybernetics).

### 2.4 RNA purification and qPCR

The mRNA levels of QKI5, NRP1 and MMP9 were quantified from total RNA using qPCR. Cells or tissues were harvested, and total RNA was isolated using TRIzol reagent (Invitrogen). Reactions for qPCR were set up using SYBR Green Master Mix (TaKaRa BIO, Shiga, Japan), and Ct values obtained for different genes of interest were normalized to levels of GAPDH. The primers used were as follows: QKI5 (forward: 5'‐AACATTAAATCACCAGCCCTTGC‐3', reverse: 5'‐CAGCTGGCGTAGGAGTACG‐3'), NRP1 (forward: 5'‐CATCTCCCGGTTACCCTCATTCTT‐3', reverse: 5'‐ GCGGCCGCCTTCATTCTC‐3'), and MMP9 (forward: 5'‐ GGGACGCAGACATCGTCATC‐3', reverse: 5'‐ GGGACGCAGACATCGTCATC‐3').

### 2.5 MTT assay

Cell proliferation was measured using the MTT assay (Invitrogen). Briefly, 3.5 × 10^3^ HTR‐8/SVneo cells per well were seeded in a 96‐well flat‐bottom plate and viability was measured every 24 h by staining with 3‐(4,5‐dimethylthiazol‐2‐yl)‐2,5‐diphenyltetrazolium bromide dye. Absorbance was measured at 450 nm using an ELx‐800 University Microplate Reader (BioTek).

### 2.6 Immunofluorescence

To visualize proteins by immunofluorescence, HTR‐8/SVneo cells were fixed 24 h post‐transfection using 4% paraformaldehyde. Cells were permeabilized with 0.1% Triton X‐100 and stained with primary antibody against QKI5 (anti‐human QKI5, 1:500, AB9904, Millipore) overnight at 4°C followed by addition of Cy3‐labeled secondary antibody ( 1:1000, ab6939, Abcam) for 50 min at room temperature. Nuclei were visualized by 4′,6‐diamidino‐2‐phenylindole staining for 10 min at room temperature. Cell proliferation was determined by staining for the Ki‐67 nuclear antigen, which is constitutively expressed throughout cell cycle progression. Images were taken using an Axio Observer microscope (Carl ZEISS) with image processing software.

### 2.7 Immunohistochemistry

Sections from the placenta were immediately fixed with formalin and embedded with paraffin. Sections of 5 μm thickness were then deparaffinized with ethanol following hydration. The sections were then washed in Tris‐buffered saline and further incubated in sodium citrate buffer containing 3% hydrogen peroxide and 50% methanol for antigen exposure. The slides were blocked using 5% blocking serum for 30 min and stained with biotin‐conjugated primary antibody against QKI5 (1:500, AB9904, Millipore) or NRP1 (1:1000, ab81321, Abcam) overnight at 4°C. Streptavidin‐conjugated secondary antibody was added along with haematoxylin for counterstaining.

### 2.8 Cell migration assay

HTR‐8/SVneo cells were starved prior to invasion assays in media containing 1% FBS. A total of 5 × 10^5^ starved cells were seeded into the upper well of a Transwell chamber (8 μm pore size; Millipore) and the lower chamber containing medium supplemented with 10% FBS served as the chemoattractant. The chambers were incubated at 37°C/5% CO_2_ for 24 h. Post‐migration cells in the lower chambers were stained with 0.1% Crystal Violet (in 20% methanol), and the number of migrated cells was quantified using five random fields per sample.

### 2.9 Wound healing assay

Pre‐seeded HTR8/SVneo cells were transfected with expression constructs or siRNA for knockdown, followed by the scratch wound healing assay 72 h later. The cells were washed in 1× phosphate‐buffered saline (PBS) and photographed. At 24 h post‐wound scratching, the cells were stained with 0.1% Crystal Violet and photographed again in the same field of view. Wound closure was calculated based on the ratio of areas uncovered by cells before and after wound scratching.

### 2.10 Luciferase reporter assay

NRP1 3'‐UTR was subcloned into a standard pGL3 luciferase reporter vector (Promega), and Renilla firefly luciferase expression was used to drive the NRP1 3'‐UTR element. The reporter construct was co‐transfected into HTR‐8/SVneo cells along with QKI5 overexpression or siRNA knockdown constructs. At 48 h post‐transfection, the cells were lysed and the lysates were incubated with luciferin. Luciferase activity as relative luciferase units (RLU) was measured using a luminometer in triplicate for each sample.

### 2.11 RNA‐protein pull‐down assay

Standard RNA pull‐down assays were conducted as follows. Cell lysates from HTR‐8/SVneo cells were incubated with biotin‐16‐UTP‐conjugated HOTAIR against 5'UTR, CDS and 3'UTR regions of NRP1, or antisense HOTAIR in immunoprecipitation (IP) buffer for 30 min at 25°C. Five μL of Dynabeads MyOne Streptavidin T1 (Invitrogen) was added to the IP mixture and incubated for 30 min. The beads containing bound RNA‐protein complexes were washed five times in 500 μL input buffer, then resuspended in protein‐loading buffer and following elution. The samples were loaded onto SDS‐PAGE gels and subjected to electrophoresis.

### 2.12 RNA immunoprecipitation (RIP) assay

The RIP assay was performed with the EZMagna RIP kit (Millipore) according to the manufacturer's instructions. Briefly, HTR‐8/SVneo cells were transfected with HOTAIR RNA antisense or control RNA oligonucleotides. At 12 h post‐transfection, the cells were harvested in ice‐cold 1× PBS and lysed in RIPA buffer. IP was conducted using RIP Ab+against QKI5 or non‐specific control rabbit IgG antibody (Millipore). The antibody‐lysate mixture was incubated overnight at 4°C with rotation. Proteins bound to the antibody were digested with proteinase K, and bound RNA was further purified from supernatant and evaluated by qPCR.

### 2.13 The PE mouse model

The PE mouse model was established using a combination of intraperitoneal injections of 125 mg/kg bodyweight of L‐NAME (eNOS inhibitor) (to mimic hypertension) and an intragastric dose of 20 mg/kg per day of resveratrol (post‐pregnancy) to female albino Wistar mice weighing at least 250 g. Control mice with no treatment and mice administered only with L‐NAME were also included. Each group consists of at least five mice. HTR‐8/SVneo cells expressing QKI5 or the respective control were transferred intravenously via tail vein injections. Urine and blood samples were collected on different gestational days (0.5, 4.5, 9, 12.5 and 18.5 days) and stored at −80°C for analysis. On gestational day 18.5, the mice were killed by cervical dislocation and the placenta and uterus were collected. All animal protocols were approved prior to experimentation.

### 2.14 Statistical analysis

Statistical analyses between data sets from at least three independent experiments were conducted using paired or unpaired Student's *t* tests to compare two groups, and one‐way analysis of variance was used to compare more than two groups. Statistics were performed with SPSS statistical software for Windows (SPSS), and a value of *P* < .5 was considered statistically significant.

## 3 RESULTS

### 3.1 Human PE placentas show reduced QKI5 expression

**FIGURE 1:**
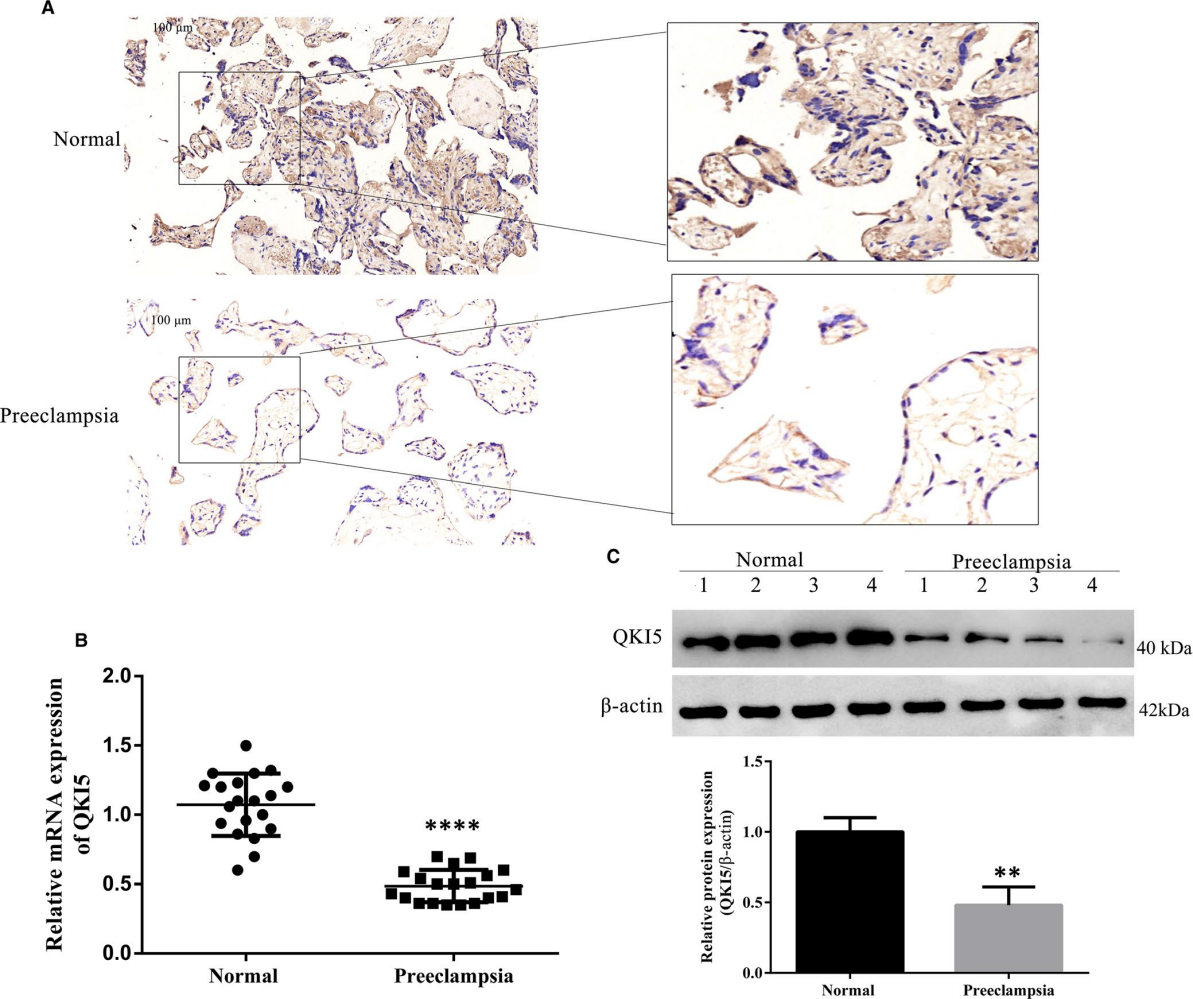
QKI5 is down‐regulated in placentas from preeclampsia patients. A, Representative images from immunohistochemistry analysis of QKI5 expression in human placentas under normal and preeclampsia conditions. Scale bar: 100 μm. B, QKI5 mRNA expression in placentas detected by qPCR. N = 20 per group. C, Representative Western blotting and densitometric quantification of QKI5 protein expression levels in four out of 20 normal and preeclampsia placentas. Shown is the mean ± standard deviation (SD) from at least three independent experiments. Statistical significance was calculated using Student's *t* test. ^****^ *P* < .0001; ^**^ *P* < .01

To examine the role of the QKI5 RNA‐binding protein in PE, we examined 20 human placental PE sections and 20 sections from healthy placentas. PE placental sections showed reduced staining for QKI5 in comparison with healthy placentas, indicating lower expression (Figure 1A). To evaluate this quantitatively, total RNA was isolated from different tissues from both groups, and mRNA levels of QKI5 were determined by qPCR. QKI5 expression was significantly reduced in PE placentas at the mRNA level when compared to healthy donors, which further confirmed the immunohistochemistry results (Figure 1B). Analysing protein expression in tissues by Western blot revealed that QKI5 expression was significantly reduced in PE tissues (Figure 1C). These initial characterizations show that QKI5 was down‐regulated in PE.

### 3.2 QKI5 expression is influenced by hypoxia and promotes cell proliferation and migration in vitro

**FIGURE 2:**
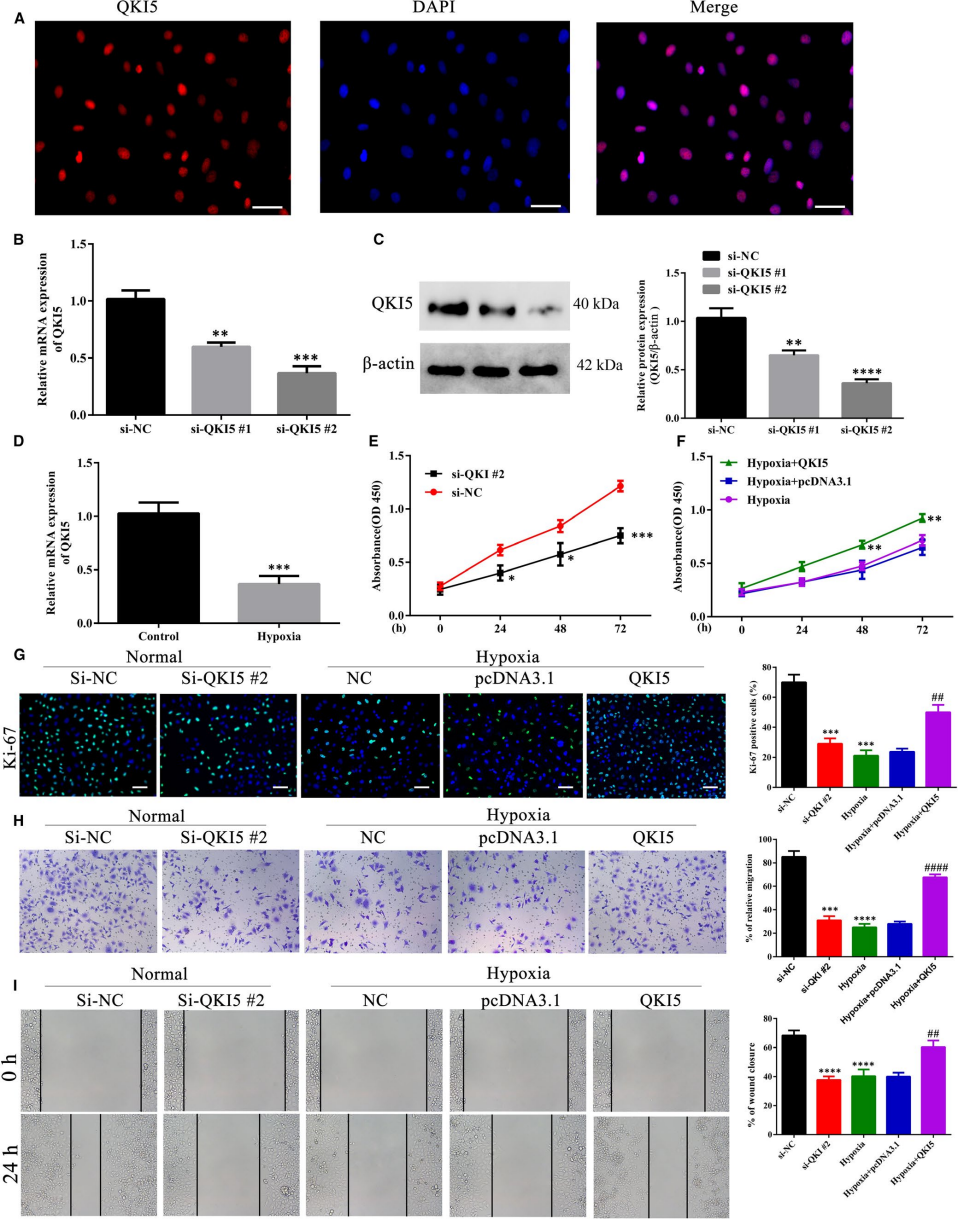
QKI5 promotes cell proliferation and migration in HTR‐8/SVneo cells during hypoxia. A, Representative micrographs of HTR‐8/SVneo cells showing the subcellular distribution of QKI5. HTR‐8/SVneo cells were fixed, permeabilized and stained with anti‐QKI5 primary antibody followed by Cy3‐labeled secondary antibody. The nuclei were stained using 4′,6‐diamidino‐2‐phenylindole (DAPI) and visualized by epifluorescence. Scale bar: 20 μm. B, Relative mRNA expression of QKI5 upon siRNA knockdown of QKI5 using two different targets. A scrambled non‐targeting siRNA (NC) was used as a control. The cells were transfected with siRNA for 24 h, then harvested for total RNA extraction, followed by cDNA synthesis and analysed by qPCR. C, Representative Western blotting (left) and densitometric quantification of cells used in panel B. D, QKI5 mRNA expression evaluated by qPCR in HTR‐8/SVneo cells cultured under normal and hypoxic conditions. E‐F, Cell proliferation of HTR‐8/SVneo cells upon knockdown or overexpression of QKI5. At 24 h post‐transfection by si‐QKI5 or the QKI5 overexpression plasmid, cell proliferation at the indicated time‐points (0 h, 24 h, 48 h and 72 h) was detected using MTT assays during normal and hypoxia conditions. Absorbance was measured at 450 nm. G, Representative merged micrographs and quantification of HTR‐8/SVneo cells upon knockdown or overexpression of QKI5 under normal or hypoxia conditions, respectively. At 24 h post‐transfection, the cells were stained with the Ki‐67 proliferation marker (green) and the nucleus was stained with DAPI (blue). At least 100 cells were counted per condition, per experiment. Scale bar: 50 μm. H‐I, Cell migration and wound healing assays. At 24 h post‐transfection using siRNA or the overexpression vector, HTR‐8/SVneo cells were added to the upper chamber of a Transwell system for migration towards the serum gradient. Cells migrating to the lower chamber 24 h post‐migration were stained using 0.1% Crystal Violet and counted to determine the migration. For the wound healing assay, a wound scratch was made in cells seeded in a 6‐well plate and images from different fields taken at the time of wound scratching and 72 h post‐wound scratching were compared to calculate the area of the wound closure. All bars show the means ± SD from at least three independent experiments. Statistical significance was calculated using Student's *t* test for comparing two groups and one‐way analysis of variance for more than two groups. ^***^ *P* < .001; ^****^ *P* < .0001 compared with the si‐NC group, ^##^ and ^####^ denote *P* < .01, *P* < .0001, respectively, compared with the hypoxia +pcDNA3.1 group

To further study the role of QKI5 in PE, we performed knockdown and overexpression studies in vitro using trophoblast HTR‐8/SVneo cells. Immunofluorescence assays revealed that the subcellular distribution of QKI5 was restricted to the cytoplasm (Figure 2A). Knockdown of QKI5 by two different siRNA targets significantly reduced mRNA expression levels of QKI5 when compared to the non‐targeting control (NC), with si‐QKI5 #2 showing a stronger effect (Figure 2B). However, both targets did not reduce mRNA expression more than twofold. This effect was also seen at the protein level with approximately 50% reduction in QKI5 when compared to si‐NC (Figure 2C). Because one of the major pathophysiological conditions of PE is hypoxia, we further tested the levels of QKI5 under hypoxic conditions (1%–2% oxygen). A twofold reduction in mRNA expression level was found when HTR‐8/SVneo cells were cultured for 24 h under hypoxic conditions, when compared to normal conditions (Figure 2D). Because siRNA #2 showed better knockdown, we chose this target for further experiments. To further assess the role of QKI5 in altering the metabolic activity and proliferative capacity of HTR‐8/SVneo cells, QKI5 was either knocked down under normal conditions or overexpressed under hypoxic conditions, then measured using the MTT procedure. The siRNA knockdown of QKI5 significantly impaired cell proliferation of HTR‐8/SVneo cells over a period of 72 h (Figure 2E), whereas under hypoxic conditions, overexpression of QKI5 significantly increased their proliferative capacities (Figure 2F). Similar results were observed when cells were stained for Ki‐67 expression to show induction of cell proliferation. QKI5 knockdown significantly impaired cell proliferation when compared with the NC, and overexpression of QKI5 alleviated this effect (Figure 2G). Because we established that QKI5 levels were modulated under hypoxic conditions, we further tested if this contributed to cell migration. Transwell experiments revealed that only 30% of HTR‐8/SVneo cells with QKI5 knockdown migrated towards a FBS gradient when compared to over 80% in the NC (Figure 2H). In addition, hypoxia also reduced cell migration to approximately 30%, which was restored to 70% when QKI5 was ectopically overexpressed (Figure 2H). Furthermore, wound healing assays also revealed that reduced QKI5 expression reduced cell migration and that this effect was reversed upon overexpression (Figure 2I). Taken together, these results showed that QKI5 was influenced by hypoxic microenvironments and was a mediator of cell proliferation and migration.

### 3.3 NRP1 is also influenced by hypoxia and is required for cell proliferation and migration

**FIGURE 3:**
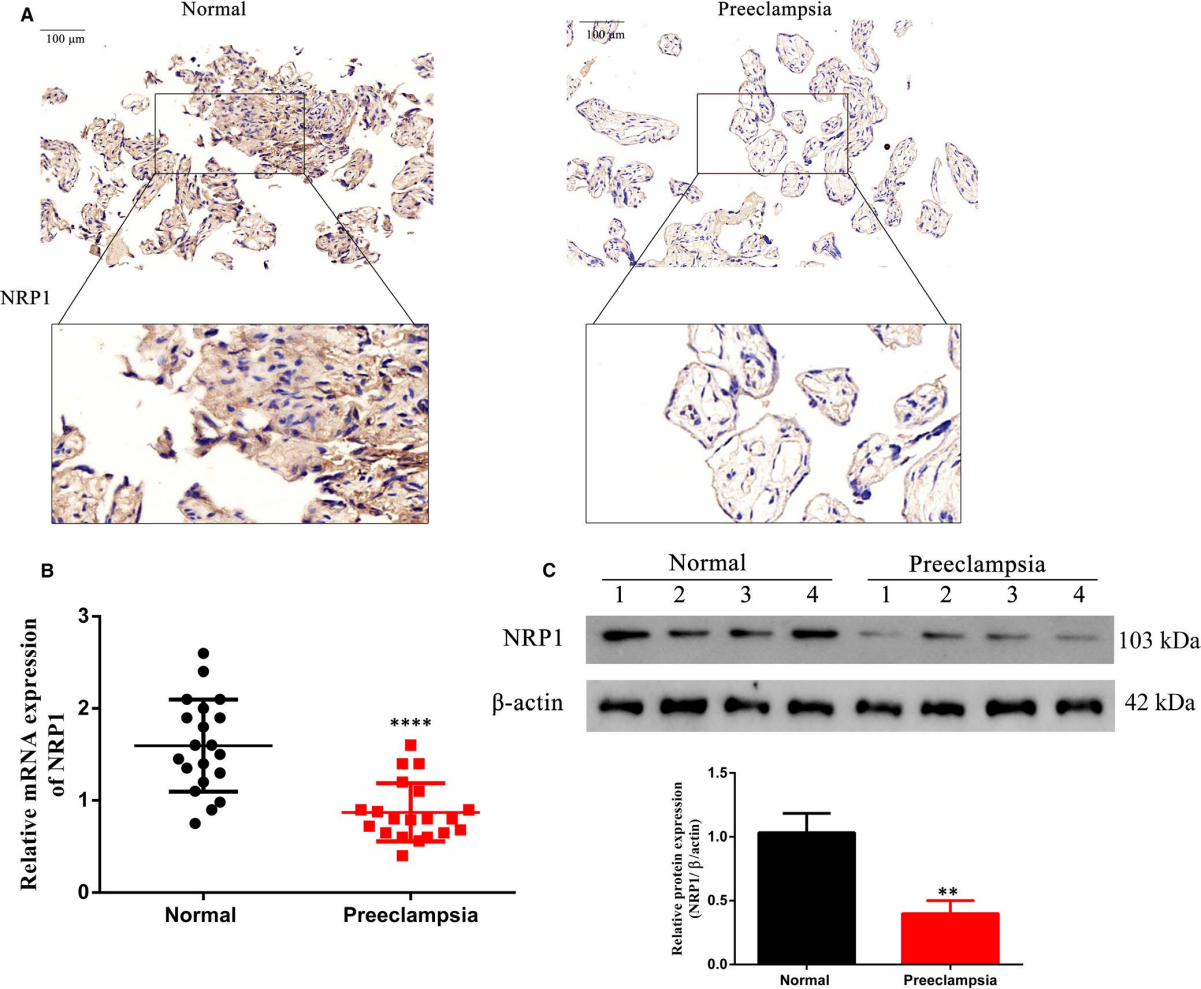
NRP1 expression is decreased in preeclampsia (PE) placentas compared with normal healthy placentas. A, Representative images from immunohistochemistry analysis of NRP1 expression in human placentas under normal and PE conditions. Scale bar: 100 μm. B, NRP1 mRNA expression in human normal placentas and PE placentas determined by qPCR. N = 20 per group. C, Representative Western blotting (left) and densitometric quantification of NRP1 protein expression in samples shown in Figure 1C. N = 20 per group. Shown are the means ± SD from at least three independent experiments. Statistical significance was calculated using Student's *t* test. ^****^ *P* < .0001; ^**^ *P* < .01

**FIGURE 4:**
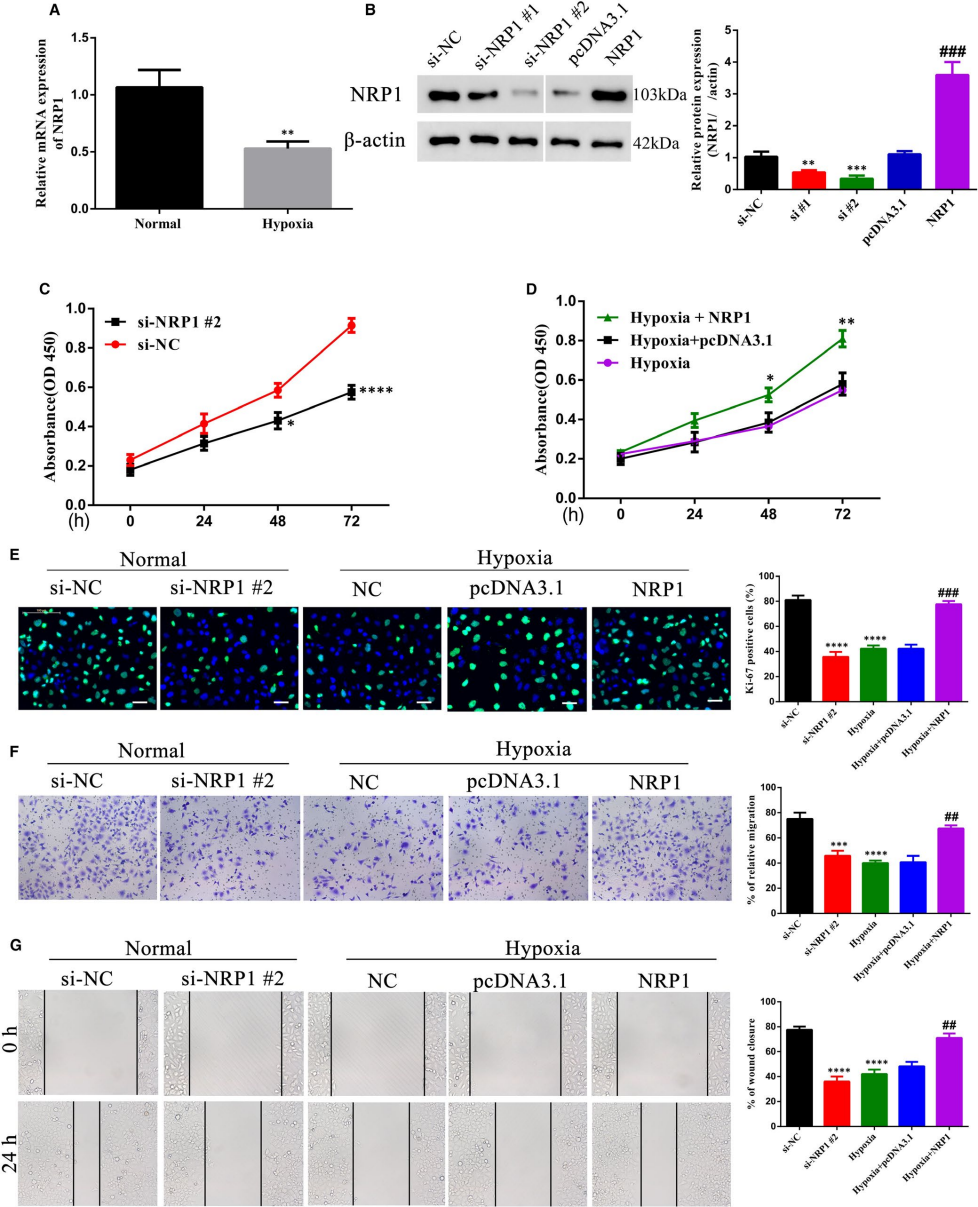
NRP1 promotes cell migration and proliferation in trophoblasts. A, Relative NRP1 mRNA levels as detected by qPCR under normal and hypoxia conditions, ^**^ *P* < .01. B, Representative Western blotting and densitometric quantification of NRP1 protein expression in HTR‐8/SVneo in non‐targeting control (NC) or NRP1 siRNA‐targeted cells. Statistics were calculated using one‐way analysis of variance. ^**^ *P* < .01; ^***^ *P* < .001 (compared with the si‐NC group); ^###^ *P* < .001 (compared with the pcDNA3.1 group). C‐D, Proliferation efficiency using the MTT assay measured as absorbance at 450 nm of trophoblast cells under normal and hypoxia conditions at 24 h post‐transfection using NRP1 siRNA or the overexpression plasmid. Statistical significance was calculated using Student's *t* test for C and one‐way analysis of variance for D. ^*^ *P* < .05; ^**^ *P* < .01; ^***^ *P* < .001. E, Representative merged micrographs and quantification of HTR‐8/SVneo NC or NRP1 siRNA‐targeted cells under normal conditions and HTR‐8/SVneo cells overexpressing NRP1 during hypoxia stained with the Ki‐67 proliferation marker (green). Nuclei were stained with DAPI (blue). At least 100 cells were counted per condition, per experiment. Scale bar: 50 μm F‐G, Cell migration efficiency. HTR‐8/SVneo NC or NRP1 siRNA‐targeted cells under normal conditions or HTR‐8/SVneo cells overexpressing NRP1 during hypoxia were assessed for their abilities to migrate in a Transwell system or by wound healing assays as mentioned in Figure 2. Shown are the means ± SD from at least three independent experiments. Statistics for panel E to G were calculated by one‐way analysis of variance. ^***^ *P* < .001; ^****^ *P* < .0001 (compared with the si‐NC group); ^##^ *P* < .01; ^###^ *P* < .001 (compared with the hypoxia +pcDNA3.1 group)

Neuropilin‐1 (NRP1) has been previously reported to be expressed at reduced levels in women with PE, along with reduced vascular endothelial growth factor (VEGF) expression.^21^, ^31^ We further confirmed this by evaluating expression levels of NRP1 in our cohort of healthy and PE tissues. NRP1 expression levels were reduced in PE placental tissues as observed by immunohistochemistry (Figure 3A), and similar reduction was also observed in NRP1 mRNA and protein expressions when compared to normal healthy tissues (Figure 3B, C). Under hypoxic conditions, NRP1 mRNA expression was reduced twofold in HTR‐8/SVneo cells (Figure 4A). Two different siRNAs targeting NRP1 reduced protein expression more than twofold in HTR‐8/SVneo cells, which could be overcome by ectopic expression of NRP1 (Figure 4B). Because the second siRNA target (si‐NRP1 #2) showed more efficient knockdown, we used it for further analysis. Similar to QKI5, knockdown of NRP1 also reduced cell proliferation as measured by the MTT assay (Figure 4C), and overexpression under hypoxic conditions significantly improved proliferation by at least twofold at 72 h (Figure 4D). Ki‐67 staining also indicated that cell proliferation was reduced to 40% upon knockdown of NRP1 or under hypoxic conditions, which was significantly improved (to 80%) when NRP1 was overexpressed (Figure 4E). Transwell migration and wound healing assays showed that cell migration was reduced to 40%‐50% upon NRP1 knockdown when compared to 80% in the NC. This effect was restored upon ectopic expression of NRP1 (Figure 4F,G). Collectively, these results suggested that NRP1 and QKI5 showed similar effects in HTR‐8/SVneo cells under hypoxic conditions.

### 3.4 NRP1 expression is directly regulated by QKI5

**FIGURE 5:**
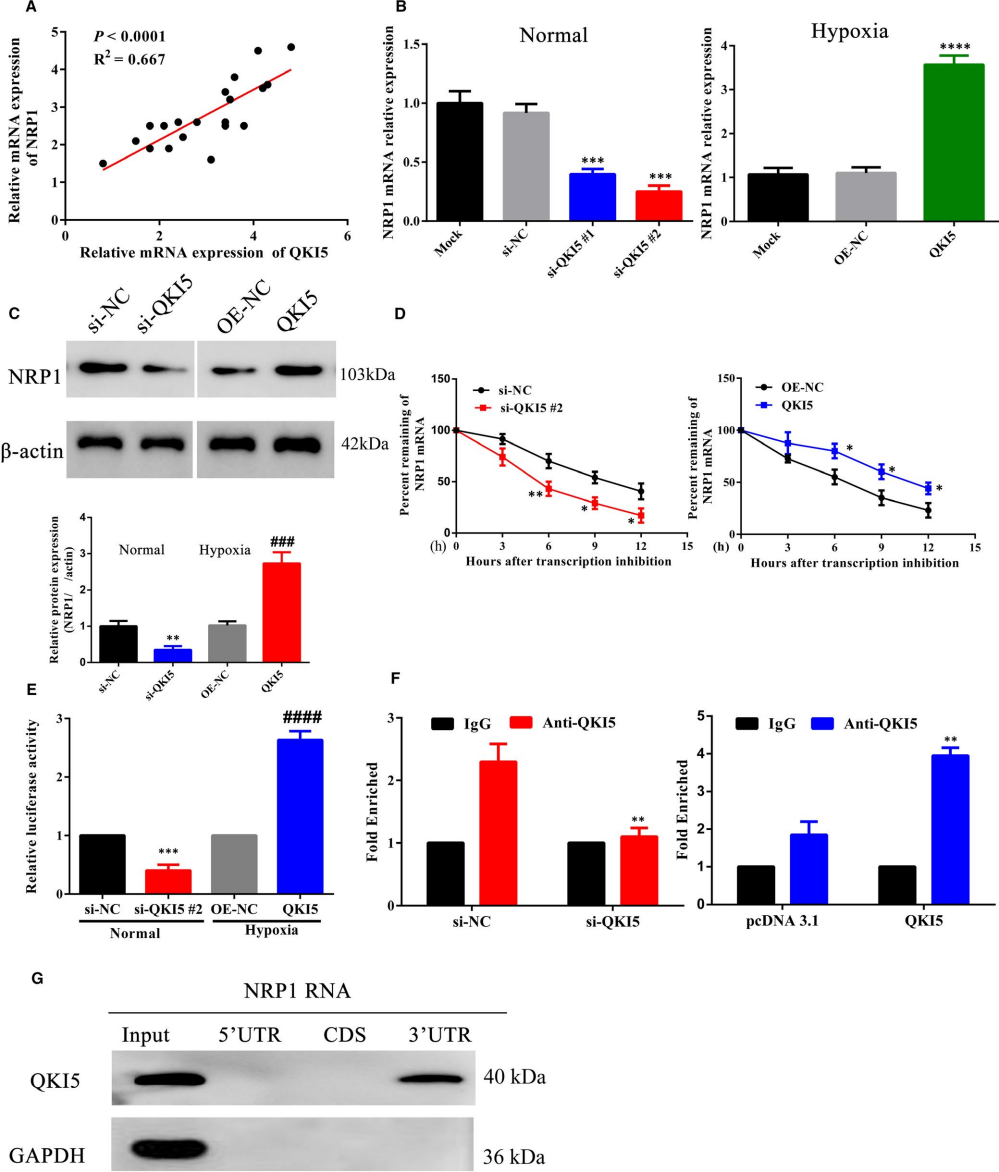
QKI5 interacts with NRP1 at the 3'UTR region. A, Scatter plot of mRNA expression levels of QKI5 and NRP1 in preeclampsia placentas. Correlations were determined using Spearman's rank correlation test (N = 20, *P <* .0001, *R* ^2^ = 0.667). B, Relative NRP1 mRNA levels as detected by qPCR in HTR‐8/SVneo cells transfected with siRNAs against QKI5 or the QKI5 overexpression vector during hypoxia. The values of mock transfection were arbitrarily set to 1. C, Representative Western blotting and densitometric quantification of NRP1 protein expressions from cells shown in panel B. D, The half‐life of NRP1 mRNA was evaluated in QKI5 knockdown or overexpression HTR‐8/SVneo cells at 0, 3, 6, 9 and 12 h post‐treatment using a transcription inhibitor. The mRNA levels at time 0 were arbitrarily set to 100%. E, The luciferase reporter assay to evaluate 3'UTR activity. NRP1 3'UTR‐mediated expression of firefly luciferase upon binding of QKI5 was determined by harvesting QKI5 knockdown or overexpression HTR‐8/SVneo cells co‐expressing the reporter construct. Cell lysates were incubated with luciferin and RLU (relative luciferase units) indicated the luciferase activity as measured using a luminometer. (F) RNA immunoprecipitation assay. Cells transfected with siRNA against QKI5 or QKI5 overexpression vector were immunoprecipitated with IgG (control) or anti‐QKI5 antibody. Bound NRP1 RNA was detected by qPCR. Values of si‐NC or the pcDNA3.1 control were arbitrarily set to 1. G, Representative Western blotting showing immunodetection of QKI5 by RNA pull‐down against different regions of NRP1 (5'UTR, CDS and 3'UTR). Glyceraldehyde 3‐phosphate dehydrogenase was used as the input control. Shown are the means ± SD from at least three independent experiments. Statistical significance was calculated using one‐way analysis of variance for all panels except D, where the two groups were compared using Student's *t* test. ^*^ *P* < .05; ^**^ *P* < .01; ^***^ *P* < .001; ^****^ *P* < .0001 (compared with the si‐NC group) and ^####^ *P* < .0001 (compared with the OE‐NC group)

Similar to its role in myeloid cell differentiation,^13^ to test further whether QKI5 altered levels of NRP1 in trophoblasts and in the context of PE, we compared their mRNA expression levels in tissue samples from PE patients. A strong correlation was observed between mRNA expression of QKI5 and NRP1 (Figure 5A). In addition, knockdown of QKI5 in HTR‐8/SVneo cells reduced mRNA levels of NRP1 by twofold, to a similar level of QKI5 previously observed (Figure 5B, Figure 2B). Under hypoxic conditions, ectopic expression of QKI5 induced more than a threefold increase in NRP1 mRNA expression (Figure 5B). Protein expression was also altered in a similar manner as mRNA levels. Targeting QKI5 by siRNA reduced NRP1 protein levels, whereas overexpression of QKI5 increased its expression (Figure 5C). To show that QKI5 directly regulated NRP1 expression, we performed mRNA decay experiments. At 6 h after addition of Actinomycin D (a transcription inhibitor), NRP1 levels were significantly reduced by approximately 50%, which decreased further over time in QKI5 knockdown cells when compared to the NC. In cells overexpressing QKI5, NRP1 mRNA levels remained significantly higher than that of NC levels, and a reduction to approximately 50% was observed only 12 h after inhibitor treatment (Figure 5D). This was further confirmed by a luciferase reporter system, where expression of Renilla firefly luciferase was driven by 3'UTR‐NRP1. Co‐transfection of the reporter construct with QKI5 siRNA or the QKI5 expression vector showed reduced expression of luciferase upon QKI5 knockdown, and increased expression upon QKI5 overexpression, indicating that QKI5 directly regulated NRP1 expression by binding to the 3'UTR region (Figure 5E). Furthermore, to confirm the direct association of QKI5 and NRP1, we performed RIP assays with control IgG or anti‐QKI5 antibody to pull down bound RNA molecules. NRP1 RNA levels were enriched twofold in anti‐QKI5 precipitates compared with the IgG control in NC cells, whereas this increase was completely lost upon QKI5 knockdown (Figure 5F). In addition, overexpression of QKI5 showed an increase in NRP1 RNA levels when precipitated with anti‐QKI5 antibody, when compared to the expression of the control vector (Figure 5F). Western blot analysis from the RNA‐protein pull‐down assay showed that QKI5 was bound only to the 3'UTR fraction of NRP1 and not to the 5'UTR or the coding sequence (CDS) (Figure 5G). In summary, these results showed that QKI5 directly regulated NRP1 expression by binding to the 3'UTR region.

### 3.5 NRP1 regulates cell migration and proliferation by MMP9 in PE

**FIGURE 6:**
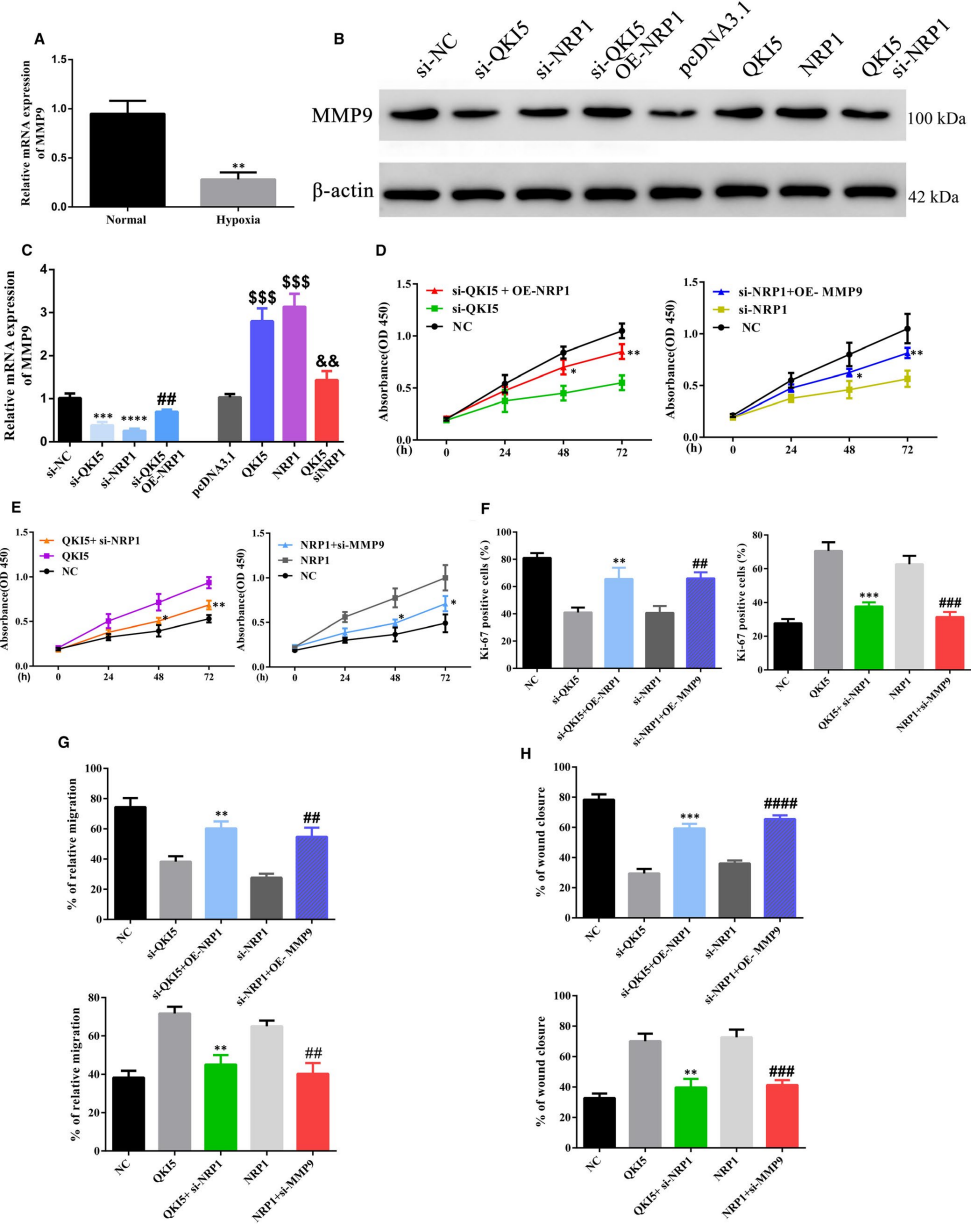
QKI5 promotes cell proliferation and migration in trophoblasts via NRP1/MMP9. A, Relative mRNA MMP9 expression levels as determined by qPCR under normal and hypoxic conditions. B, C, Representative Western blotting and quantification of MMP9 protein expressions under different conditions in HTR‐8/SVneo cells. D‐E, Cell proliferation was determined using the MTT assay. At 24 h post‐transfection, the cells were stained with the MTT dye, and the absorbance was measured at 450 nm. F, Representative merged micrographs and quantitative analysis of cell proliferation by Ki‐67 staining. Nuclei were stained using DAPI. At least 100 cells were counted per condition, per experiment (G‐H) Cell migration and invasion assays. The relative percentage migration and invasion were calculated using the Transwell migration or wound healing assays in HTR‐8/SVneo cells under different conditions. Statistical significance was calculated using one‐way analysis of variance for all panels except A, where the two groups were compared using Student's *t* test. ^**^ *P* < .01; ^***^ *P* < .001; ^****^ *P* < .0001 (compared with the non‐targeting group); ^##^ *P* < .01; ^###^ *P* < .001; ^####^ *P* < .0001 (compared with the NRP1 or si‐NRP1 group); ^$$$^ *P* < .001 (compared with the pcDNA3.1 group); in panel C, ^&&^ *P* < .01 (compared with the NRP1 group). Shown are the means ± SD from at least three independent experiments

In addition to the effects of QKI5 on NRP1 expression, we evaluated its role in targeting other factors involved in this signalling pathway. Matrix metalloproteinase‐9 (MMP9) functions in releasing growth factors to trigger VEGF‐R/NRP1 pathway. We found that MMP9 mRNA expression was reduced more than twofold under hypoxic conditions in HTR‐8/SVneo cells (Figure 6A). Protein levels of MMP9 were also decreased upon QKI5 and NRP1 knockdown, and this effect was overcome upon NRP1 overexpression (Figure 6B). In a similar manner, QKI5 and NRP1 overexpression increased levels of MMP9 expression, and this effect was reduced after knockdown of NRP1 knockdown (Figure 6B). Relative mRNA levels were also similar to protein levels, whereupon knockdown of QKI5 and NRP1, MMP9 mRNA levels were significantly reduced, while this effect was rescued by NRP1 overexpression (Figure 6C). Similarly, QKI5 and NRP1 overexpression significantly increased MMP9 mRNA expression, and this effect was reduced upon NRP1 knockdown (Figure 6C). The MTT assay, which measures cell proliferation, showed that QKI5 knockdown significantly reduced cell proliferation as previously observed, and this effect was improved 48 h after overexpression of NRP1 (Figure 6D). In addition, overexpression of MMP9 in NRP1 knockdown cells also significantly improved cell proliferation 48 h after knockdown (Figure 6D). Similar experiments with siRNA knockdown of NRP1 or MMP9 along with overexpression of QKI5 or NRP1 significantly reduced cell proliferation compared with overexpression alone (Figure 6E). Staining after NRP1 and MMP9 overexpression or knockdown of cells with Ki‐67 showed similar results, where overexpression of NRP1 or MMP9 significantly improved cell proliferation, whereas knockdown reduced this effect (Figure 6F). Analysing cell migration by using the Transwell and wound healing assays revealed that upon overexpression of NRP1 or MMP9 along with siRNA knockdown of QKI5 or NRP1, the migration or wound closure was significantly improved by 20%–30% compared with knockdown conditions (Figure 6G,H; upper panel). However, knockdown of NRP1 and MMP9 together with overexpression of QKI5 and NRP1 significantly reduced migration and wound closure by 20%‐30% (Figure 6G,H; lower panel). Together, these results indicated that MMP9 was positively regulated by NRP1, which affected cell proliferation and migration in vitro.

### 3.6 Increased QKI5 expression reduces the severity of PE symptoms in an in vivo mouse model

**FIGURE 7:**
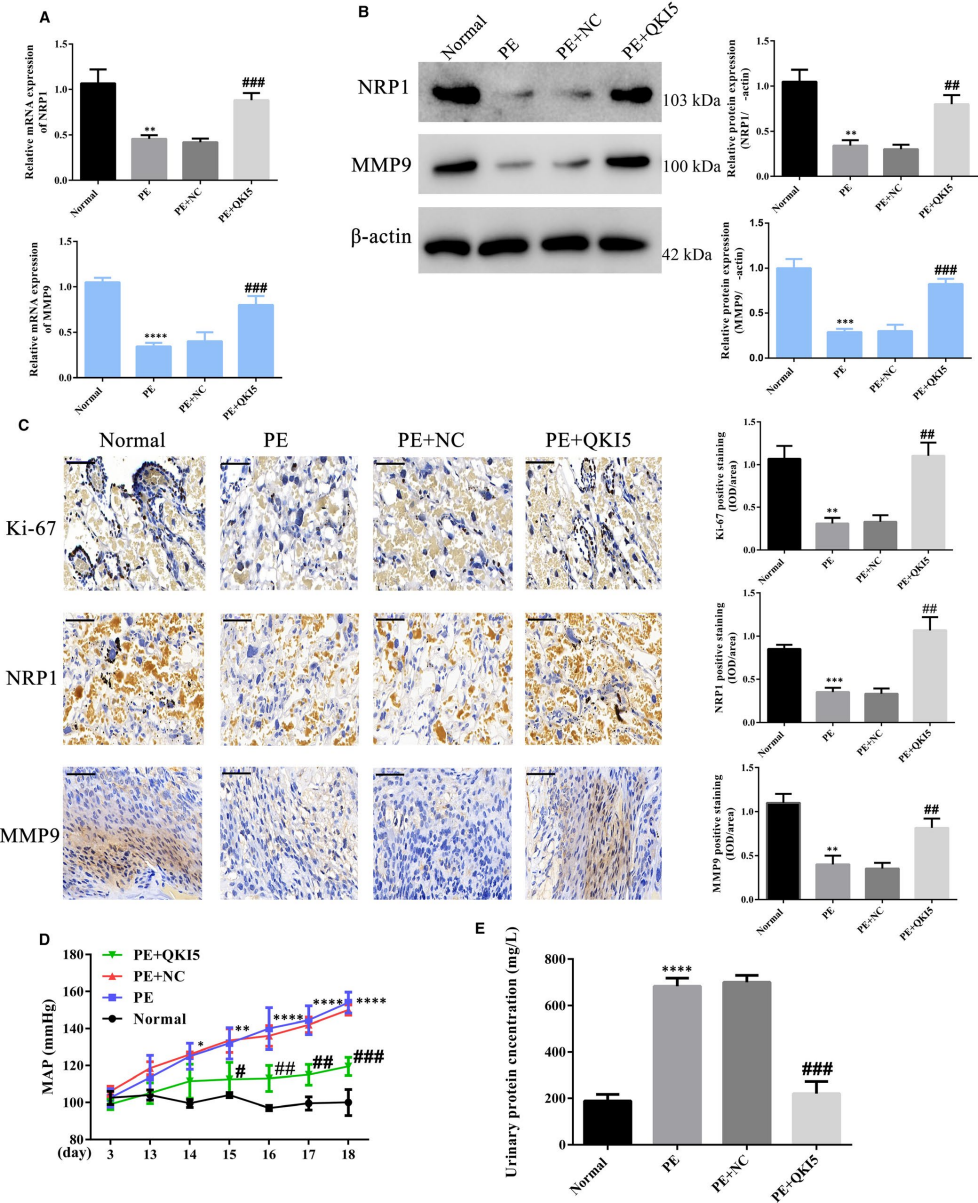
QKI5 overexpression improves preeclampsia (PE)‐related effects in vivo by regulating NRP1/MMP9 expression. A, Relative mRNA expression of NRP1 and MMP9 determined by qPCR from placental tissues obtained from mice belonging to different groups: The saline‐treated group (normal), the L‐NAME‐treated group (PE), the group with L‐NAME‐treated mice bearing adoptively transferred NC HTR‐8/SVneo cells (PE+NC) and L‐NAME‐treated mice bearing QKI5 expressing HTR‐8/SVneo cells (PE+QKI5). B, Representative Western blotting and densitometric quantification of NRP1 and MMP9 protein expressions relative to the β‐actin loading control. C, Representative immunohistochemistry images and corresponding integrated absorbance values from stained placental tissues indicating proliferation by Ki‐67 staining, and NRP1 and MMP9 expressions. D, The blood pressure indicated by mean arterial pressure in different groups over 18 gestational days. E, Total protein concentration determined in urine samples from day 18.5. Shown are the means ± SD from five mice from each group. Statistical significance was calculated using one‐way analysis of variance. ^**^ *P* < .01; ^***^ *P* < .001; ^****^ *P* < .0001 (compared with the non‐targeting group); ^#^ *P* < .05; ^##^ *P* < .01; ^###^ *P* < .001 (compared with the PE+NC group)

We next sought to test these effects in an in vivo mouse model for PE. ^36^ Normal mice with no treatment (Normal), PE mice with or without adoptive transfer of NC HTR‐8/SVneo cells (PE+NC or PE) and PE mice adoptively transferred with QKI5 overexpression cells (PE+QKI5) were used. Placental tissues from PE mice harvested towards the end of gestation showed reduced NRP1 and MMP9 mRNA expressions, whereas overexpression of QKI5 restored NRP1 levels to that of normal mice (Figure 7A). Similarly, protein expression analysis revealed that in PE mice, both NRP1 and MMP9 levels were significantly reduced, and this effect was reversed when QKI5 was overexpressed (Figure 7B). Immunohistochemistry analyses of tissue samples stained with cell proliferation dyes, Ki‐67, NRP1 or MMP9, showed that cell proliferation was significantly reduced in PE and PE+NC tissues, whereas upon QKI5 overexpression, the levels were increased to levels comparable with normal mice (Figure 7C). In addition, continuous monitoring of blood pressure and urine protein concentration showed a steady increase in blood pressure over time in the PE mice groups, and this effect was significantly reduced upon QKI5 overexpression (Figure 7D). A threefold increase in total urine protein concentration was also observed in PE mice, and this effect was reduced to levels comparable with normal mice upon QKI5 overexpression (Figure 7E). Taken together, these results showed that QKI5 promoted cell proliferation, possibly via a NRP1/MMP9‐dependent manner in an in vivo PE mouse model.

## 4 DISCUSSION

During early stages of pregnancy, invasion of trophoblasts through the uterine wall is an important first step in placentation. These trophoblasts lining the uterus are responsible for nourishing the uterine environment with oxygen and sufficient nutrients. Defects in these initial processes due to hypoxia and insufficient arterial blood flow cause severe complications in pregnancy. Hypoxic trophoblasts release proinflammatory cytokines to induce systemic inflammation and apoptosis.^30^ However, hypoxic environments also reprogram cell fate by causing changes in transcriptional regulation and down‐regulation of intrinsic growth factors that negatively regulate cell survival and proliferation. In line with this, we observed that RNA‐binding protein QKI5 regulated expression of two key targets involved in VEGF signalling under hypoxia: NRP1 and MMP9. QKI5‐dependent functional defect in trophoblast development and migration was alleviated upon restoring one of these factors indicating dual control of a single pathway.

QKI proteins play an important role in cell growth and differentiation. For example, QKI5 regulates expression of factors involved in the development of red blood cells by affecting RNA stability and interfering with protein translation.^32^, ^33^ In placental tissues from PE patients, we observed a significant down‐regulation in levels of QKI5. Similarly, under hypoxia, HTR‐8/SVneo trophoblast cells showed reduced expression of QKI5 that resulted in decreased cell proliferation, migration and invasion. Cell proliferation and migration are controlled by several pathways including growth factor and chemokine signalling pathways, and RNA‐binding proteins can act as both direct and indirect targets. Indirect targeting is mediated via binding with regulatory microRNAs to affect gene expression.^34^ However, they can also directly affect expression of growth factors, receptors and downstream signalling molecules.

One of the major signalling pathways required for trophoblast migration and invasion is the VEGF‐R/NRP1 pathway.^35^ It is activated upon binding of soluble VEGF released from the extracellular matrix by matrix metalloproteinases (MMPs). Among the different forms of VEGF‐R, VEGF‐A interacts with the NRP1 co‐receptor to promote cell migration and angiogenesis.^36^ A previous study showed that reduced trophoblast development observed in PE placentas correlated with down‐regulation of VEGF and NRP1 expression.^21^ We also observed a reduction in NRP1 expression at the mRNA and protein levels in placental tissues from PE patients, which further correlated with QKI5 expression. Furthermore, knockdown and overexpression of NRP1 in HTR‐8/SVneo cells showed that NRP1 was also essential for cell proliferation and migration during hypoxia. RIP assays revealed that QKI5 directly interacted with NRP1 mRNA, and RNA pull‐down assays using different domains of NRP1 showed that QKI5 specifically bound to the 3'UTR region. This indicates that QKI5 does not interfere with microRNAs to regulate expression of NRP1 but directly associates with it to affect mRNA stability and reduce translation efficiency.^37^

We further identified that QKI5 did not exclusively target NRP1 to interfere with the VEGF‐R/NRP1 pathway but also affected expression of MMP9 in HTR‐8/SVneo cells under hypoxia. MMPs regulate VEGF‐R/NRP1 signalling by promoting the release of matrix‐bound VEGF, and their role in trophoblast invasion has been described.^23^, ^38^, ^39^ In cancers, increased levels of MMP9 has been associated with tumorigenesis and metastasis;^40^, ^41^ however, in PE their expression is down‐regulated.^29^ Although we did not elucidate the mechanism of reduced MMP9 expression in the presence of QKI5, we observed that its functional effect on cell proliferation and invasion was overcome upon expression of NRP1. A previous report studying pregnancy‐related hypertensive disorders using placental tissues showed that MMP9 expression was significantly reduced in a severe hypertensive group when compared to healthy patients.^42^ This further suggests that alteration in MMP9 levels as a result of comorbidities can be independent of other failures in growth factor signalling observed in PE. QKI5 emerges as a common determinant that can regulate expression of both proteins that are key promoters of cellular development.

The implications of QKI5‐mediated effects in vivo during the progression of PE was studied using an adoptive transfer mouse model where received cells overexpressing QKI5. This model was chosen over the STOX‐1 model that recapitulates early PE maternal syndrome and other available PE models as it proved relevant to study both placental pathology and symptoms related to PE such as rise in blood pressure and increased urine/creatine levels.^43^, ^44^ Placental tissues harvested towards the end of gestation revealed a significant increase in cell proliferation in the QKI5 treatment group. We also observed a significant decrease in PE symptoms such as blood pressure elevation and total protein concentration in the urine. Future studies using other mouse models including endocrine gland‐derived VEGF (EG‐VEGF) or MMP9‐null mice model would provide more insight into the relevance of QKI5 in PE pathogenesis.^45^, ^46^, ^47^

These results together identified a novel mechanism responsible for trophoblast dysregulation observed in PE, where QKI5‐mediated regulation of NRP1/MMP9 expression significantly altered cell proliferation and migration.

## CONFLICT OF INTEREST

The authors declare that they have no competing interests.

## AUTHOR CONTRIBUTION

**Xingyu Yang:** Formal analysis (equal); Software (equal); Writing‐original draft (equal). **Dan Chen:** Data curation (equal); Methodology (equal); Software (equal). **Biwei He:** Investigation (equal); Software (equal); Visualization (equal). **Weiwei Cheng:** Conceptualization (equal); Supervision (equal); Writing‐review & editing (equal).

## Data Availability

The data that support the findings of this study are available from the corresponding author upon reasonable request.

## References

[1] Wilkerson RG , Ogunbodede AC . Hypertensive disorders of pregnancy. Emerg Med Clin North Am. 2019;37(2):301‐316. 30940374 10.1016/j.emc.2019.01.008

[2] Say L , Chou D , Gemmill A , et al. Global causes of maternal death: a WHO systematic analysis. Lancet Glob Health. 2014;2(6):e323‐e333. 25103301 10.1016/S2214-109X(14)70227-X

[3] Liang J , Li X , Kang C , et al. Maternal mortality ratios in 2852 Chinese counties, 1996–2015, and achievement of Millennium Development Goal 5 in China: a subnational analysis of the Global Burden of Disease Study 2016. Lancet. 2019;393(10168):241‐252. 30554785 10.1016/S0140-6736(18)31712-4 PMC6336935

[4] Pennington KA , Schlitt JM , Jackson DL , Schulz LC , Schust DJ . Preeclampsia: multiple approaches for a multifactorial disease. Dis Model Mech. 2012;5(1):9‐18. 22228789 10.1242/dmm.008516 PMC3255538

[5] Silasi M , Cohen B , Karumanchi SA , Rana S . Abnormal placentation, angiogenic factors, and the pathogenesis of preeclampsia. Obstet Gynecol Clin North Am. 2010;37(2):239‐253. 20685551 10.1016/j.ogc.2010.02.013

[6] Khong TY , De Wolf F , Robertson WB , Brosens I . Inadequate maternal vascular response to placentation in pregnancies complicated by pre‐eclampsia and by small‐for‐gestational age infants. Br J Obstet Gynaecol. 1986;93(10):1049‐1059. 3790464 10.1111/j.1471-0528.1986.tb07830.x

[7] Redman CW , Sargent IL . Placental stress and pre‐eclampsia: a revised view. Placenta. 2009;30(Suppl A):S38‐42. 19138798 10.1016/j.placenta.2008.11.021

[8] Wu D , Chen X , Wang L , Chen F , Cen H , Shi L . Hypoxia‐induced microRNA‐141 regulates trophoblast apoptosis, invasion, and vascularization by blocking CXCL12beta/CXCR2/4 signal transduction. Biomed Pharmacother. 2019;116:108836. 31004838 10.1016/j.biopha.2019.108836

[9] Albers RE , Kaufman MR , Natale BV , et al. Trophoblast‐specific expression of Hif‐1alpha results in preeclampsia‐like symptoms and fetal growth restriction. Sci Rep. 2019;9(1):2742. 30808910 10.1038/s41598-019-39426-5 PMC6391498

[10] Hung TH , Burton GJ . Hypoxia and reoxygenation: a possible mechanism for placental oxidative stress in preeclampsia. Taiwan J Obstet Gynecol. 2006;45(3):189‐200. 17175463 10.1016/S1028-4559(09)60224-2

[11] Kaufmann P , Black S , Huppertz B . Endovascular trophoblast invasion: implications for the pathogenesis of intrauterine growth retardation and preeclampsia. Biol Reprod. 2003;69(1):1‐7. 12620937 10.1095/biolreprod.102.014977

[12] de Bruin RG , Shiue L , Prins J , et al. Quaking promotes monocyte differentiation into pro‐atherogenic macrophages by controlling pre‐mRNA splicing and gene expression. Nat Commun. 2016;7:10846. 27029405 10.1038/ncomms10846 PMC4821877

[13] Zhang H , Prins J , Vreeken D , et al. Comprehensive analysis of neuronal guidance cue expression regulation during monocyte‐to‐macrophage differentiation reveals post‐transcriptional regulation of semaphorin7A by the RNA‐binding protein quaking. Innate Immun. 2021;27(2):118‐132. 33241976 10.1177/1753425920966645 PMC7882812

[14] Shi F , Wei D , Zhu Z , et al. The RNA‐binding protein QKI suppresses tumorigenesis of clear cell renal cell carcinoma by regulating the expression of HIF‐1alpha. J Cancer. 2020;11(6):1359‐1370. 32047543 10.7150/jca.36083 PMC6995368

[15] Cochrane A , Kelaini S , Tsifaki M , et al. Quaking is a key regulator of endothelial cell differentiation, neovascularization, and angiogenesis. Stem Cells. 2017;35(4):952‐966. 28207177 10.1002/stem.2594 PMC5396345

[16] Yang C , Eleftheriadou M , Kelaini S , et al. Targeting QKI‐7 in vivo restores endothelial cell function in diabetes. Nat Commun. 2020;11(1):3812. 32732889 10.1038/s41467-020-17468-y PMC7393072

[17] Coomer AO , Black F , Greystoke A , Munkley J , Elliott DJ . Alternative splicing in lung cancer. Biochim Biophys Acta Gene Regul Mech. 2019;1862(11–12):194388. 31152916 10.1016/j.bbagrm.2019.05.006

[18] Wang JZ , Fu X , Fang Z , et al. QKI‐5 regulates the alternative splicing of cytoskeletal gene ADD3 in lung cancer. J Mol Cell Biol. 2020. Epub ahead of print. 10.1093/jmcb/mjaa063 PMC8373271 33196842

[19] Pratt A , Da Silva CF , Borg AJ , Kalionis B , Keogh R , Murthi P . Placenta‐derived angiogenic proteins and their contribution to the pathogenesis of preeclampsia. Angiogenesis. 2015;18(2):115‐123. 25433512 10.1007/s10456-014-9452-3

[20] Lanahan A , Zhang X , Fantin A , et al. The neuropilin 1 cytoplasmic domain is required for VEGF‐A‐dependent arteriogenesis. Dev Cell. 2013;25(2):156‐168. 23639442 10.1016/j.devcel.2013.03.019 PMC3774154

[21] Xu X , Yang XY , He BW , Yang WJ , Cheng WW . Placental NRP1 and VEGF expression in pre‐eclamptic women and in a homocysteine‐treated mouse model of pre‐eclampsia. Eur J Obstet Gynecol Reprod Biol. 2016;196:69‐75. 26708340 10.1016/j.ejogrb.2015.11.017

[22] Mannello F , Tonti GA , Bagnara GP , Papa S . Role and function of matrix metalloproteinases in the differentiation and biological characterization of mesenchymal stem cells. Stem Cells. 2006;24(3):475‐481. 16150919 10.1634/stemcells.2005-0333

[23] Anacker J , Segerer SE , Hagemann C , et al. Human decidua and invasive trophoblasts are rich sources of nearly all human matrix metalloproteinases. Mol Hum Reprod. 2011;17(10):637‐652. 21565864 10.1093/molehr/gar033

[24] Alexander CM , Hansell EJ , Behrendtsen O , et al. Expression and function of matrix metalloproteinases and their inhibitors at the maternal‐embryonic boundary during mouse embryo implantation. Development. 1996;122(6):1723‐1736. 8674412 10.1242/dev.122.6.1723

[25] Shi F , Shang L , Pan BQ , et al. Calreticulin promotes migration and invasion of esophageal cancer cells by upregulating neuropilin‐1 expression via STAT5A. Clin Cancer Res. 2014;20(23):6153‐6162. 25231404 10.1158/1078-0432.CCR-14-0583

[26] Zhang Y , Chen C , Yao Q , Li M . ZIP4 upregulates the expression of neuropilin‐1, vascular endothelial growth factor, and matrix metalloproteases in pancreatic cancer cell lines and xenografts. Cancer Biol Ther. 2010;9(3):236‐242. 20023433 10.4161/cbt.9.3.10749 PMC2896555

[27] Chen J , Khalil RA . Matrix Metalloproteinases in normal pregnancy and preeclampsia. Prog Mol Biol Transl Sci. 2017;148:87‐165. 28662830 10.1016/bs.pmbts.2017.04.001 PMC5548443

[28] Espino YSS , Flores‐Pliego A , Espejel‐Nunez A , et al. New insights into the role of matrix metalloproteinases in preeclampsia. Int J Mol Sci. 2017;18(7):1448. 10.3390/ijms18071448 PMC5535939 28726716

[29] Zhang Y , Li P , Guo Y , Liu X , Zhang Y . MMP‐9 and TIMP‐1 in placenta of hypertensive disorder complicating pregnancy. Exp Ther Med. 2019;18(1):637‐641. 31258700 10.3892/etm.2019.7591 PMC6566117

[30] Cheng SB , Nakashima A , Huber WJ , et al. Pyroptosis is a critical inflammatory pathway in the placenta from early onset preeclampsia and in human trophoblasts exposed to hypoxia and endoplasmic reticulum stressors. Cell Death Dis. 2019;10(12):927. 31804457 10.1038/s41419-019-2162-4 PMC6895177

[31] Herzog B , Pellet‐Many C , Britton G , Hartzoulakis B , Zachary IC . VEGF binding to NRP1 is essential for VEGF stimulation of endothelial cell migration, complex formation between NRP1 and VEGFR2, and signaling via FAK Tyr407 phosphorylation. Mol Biol Cell. 2011;22(15):2766‐2776. 21653826 10.1091/mbc.E09-12-1061 PMC3145551

[32] Wang F , Song W , Zhao H , et al. The RNA‐binding protein QKI5 regulates primary miR‐124‐1 processing via a distal RNA motif during erythropoiesis. Cell Res. 2017;27(3):416‐439. 28244490 10.1038/cr.2017.26 PMC5339841

[33] Teplova M , Hafner M , Teplov D , Essig K , Tuschl T , Patel DJ . Structure‐function studies of STAR family Quaking proteins bound to their in vivo RNA target sites. Genes Dev. 2013;27(8):928‐940. 23630077 10.1101/gad.216531.113 PMC3650229

[34] Kim EJ , Kim JS , Lee S , et al. QKI, a miR‐200 target gene, suppresses epithelial‐to‐mesenchymal transition and tumor growth. Int J Cancer. 2019;145(6):1585‐1595. 31026342 10.1002/ijc.32372

[35] Xiao Z , Li S , Yu Y , et al. VEGF‐A regulates sFlt‐1 production in trophoblasts through both Flt‐1 and KDR receptors. Mol Cell Biochem. 2018;449(1–2):1‐8. 29497919 10.1007/s11010-018-3337-5

[36] Becker PM , Waltenberger J , Yachechko R , et al. Neuropilin‐1 regulates vascular endothelial growth factor‐mediated endothelial permeability. Circ Res. 2005;96(12):1257‐1265. 15920019 10.1161/01.RES.0000171756.13554.49

[37] Wang Y , Vogel G , Yu Z , Richard S . The QKI‐5 and QKI‐6 RNA binding proteins regulate the expression of microRNA 7 in glial cells. Mol Cell Biol. 2013;33(6):1233‐1243. 23319046 10.1128/MCB.01604-12 PMC3592017

[38] Bergers G , Brekken R , McMahon G , et al. Matrix metalloproteinase‐9 triggers the angiogenic switch during carcinogenesis. Nat Cell Biol. 2000;2(10):737‐744. 11025665 10.1038/35036374 PMC2852586

[39] Belotti D , Paganoni P , Manenti L , et al. Matrix metalloproteinases (MMP9 and MMP2) induce the release of vascular endothelial growth factor (VEGF) by ovarian carcinoma cells: implications for ascites formation. Cancer Res. 2003;63(17):5224‐5229. 14500349

[40] Kleiner DE , Stetler‐Stevenson WG . Matrix metalloproteinases and metastasis. Cancer Chemother Pharmacol. 1999;43(Suppl):S42‐51. 10357558 10.1007/s002800051097

[41] Funahashi Y , Shawber CJ , Sharma A , Kanamaru E , Choi YK , Kitajewski J . Notch modulates VEGF action in endothelial cells by inducing Matrix Metalloprotease activity. Vasc Cell. 2011;3(1):2. 21349159 10.1186/2045-824X-3-2 PMC3039832

[42] Palei AC , Sandrim VC , Amaral LM , et al. Matrix metalloproteinase‐9 polymorphisms affect plasma MMP‐9 levels and antihypertensive therapy responsiveness in hypertensive disorders of pregnancy. Pharmacogenomics J. 2012;12(6):489‐498. 21769110 10.1038/tpj.2011.31

[43] Sones JL , Davisson RL . Preeclampsia, of mice and women. Physiol Genomics. 2016;48(8):565‐572. 27260843 10.1152/physiolgenomics.00125.2015 PMC5005458

[44] Miralles F , Collinot H , Boumerdassi Y , et al. Long‐term cardiovascular disorders in the STOX1 mouse model of preeclampsia. Sci Rep. 2019;9(1):11918. 31417152 10.1038/s41598-019-48427-3 PMC6695383

[45] Dubois B , Arnold B , Opdenakker G . Gelatinase B deficiency impairs reproduction. J Clin Invest. 2000;106(5):627‐628. 10974013 10.1172/JCI10910 PMC381291

[46] Doridot L , Passet B , Mehats C , et al. Preeclampsia‐like symptoms induced in mice by fetoplacental expression of STOX1 are reversed by aspirin treatment. Hypertension. 2013;61(3):662‐668. 23357179 10.1161/HYPERTENSIONAHA.111.202994

[47] Plaks V , Rinkenberger J , Dai J , et al. Matrix metalloproteinase‐9 deficiency phenocopies features of preeclampsia and intrauterine growth restriction. Proc Natl Acad Sci U S A. 2013;110(27):11109‐11114. 23776237 10.1073/pnas.1309561110 PMC3704020

